# Do you hear where I hear?: isolating the individualized sound localization cues

**DOI:** 10.3389/fnins.2014.00370

**Published:** 2014-12-01

**Authors:** Griffin D. Romigh, Brian D. Simpson

**Affiliations:** Air Force Research LaboratoryDayton, OH, USA

**Keywords:** head-related transfer function, spatial hearing, individual differences, auditory display

## Abstract

It is widely acknowledged that individualized head-related transfer function (HRTF) measurements are needed to adequately capture all of the 3D spatial hearing cues. However, many perceptual studies have shown that localization accuracy in the lateral dimension is only minimally decreased by the use of non-individualized head-related transfer functions. This evidence supports the idea that the individualized components of an HRTF could be isolated from those that are more general in nature. In the present study we decomposed the HRTF at each location into average, lateral and intraconic spectral components, along with an ITD in an effort to isolate the sound localization cues that are responsible for the inter-individual differences in localization performance. HRTFs for a given listener were then reconstructed systematically with components that were both individualized and non-individualized in nature, and the effect of each modification was analyzed via a virtual localization test where brief 250 ms noise bursts were rendered with the modified HRTFs. Results indicate that the cues important for individualization of HRTFs are contained almost exclusively in the intraconic portion of the HRTF spectra and localization is only minimally affected by introducing non-individualized cues into the other HRTF components. These results provide new insights into what specific inter-individual differences in head-related acoustical features are most relevant to sound localization, and provide a framework for how future human-machine interfaces might be more effectively generalized and/or individualized.

## 1. Introduction

It has long been the desire of auditory scientists to discover and map how specific physical features of the sound arriving at the two ears translate to distinct locations in perceptual space. While much progress has been made toward accomplishing this feat, the highly-individual nature of high-frequency spectral cues used for localization in the vertical and front-back dimensions has thwarted most efforts to create a universally accepted feature-based model for localization in these dimensions.

All of the physical cues available to a listener for making spatial judgments are captured in a listener's head-related transfer function, which describes the transformation a sound undergoes as it travels from a specific location in space, interacts with the listener's head, shoulders, and outer ears and arrives at a listener's eardrums (Mehrgardt and Mellert, 1977). These transfer functions can be calculated for a specific sound source direction by outfitting a listener with binaural microphones and recording the arrival of a known signal presented from the desired location (Mehrgardt and Mellert, 1977; Wightman and Kistler, 1989a). Once measured for an individual, this transfer function can be used to impart spatial information on an arbitrary single-channel sound to create the perceptual illusion that the sound originates from an actual position out in space when presented over headphones (Wightman and Kistler, 1989b; Bronkhorst, 1995; Brungart et al., 2009).

While virtual auditory displays (VADs) based on this technology have been employed in many applications including entertainment, gaming, virtual reality (Travis, 1996) and navigational aids for pilots (Simpson et al., 2007), high fidelity performance, or more specifically accurate localization in the vertical and front-back dimensions, requires that the HRTF be measured on the specific user utilizing the display, limiting their widespread implementation. Several authors have shown that when VADs use HRTFs measured on a different individual or acoustic mannequin, localization performance is severely degraded, resulting in especially poor elevation localization and frequent confusions about the front-back hemisphere of the target sound (Wenzel et al., 1993; Middlebrooks, 1999a; Brungart and Romigh, 2009).

The cues believed to be responsible for localization in these dimensions are found in the high-frequency (above 4 kHz) region of the right and left monaural HRTF magnitude spectra (Hebrank and Wright, 1974; Asano et al., 1990). This region of the HRTF is also impacted greatly by the effect of head shadow on the contralateral ear, a feature that leads to the interaural level cue used for lateral location judgments (Blauert, 1997). This means that the physical cues for both lateral localization judgments and vertical and front-back judgments are combined in the high-frequency HRTF spectrum. While much work has been done to better understand localization cues in the vertical and front-back dimensions (Blauert, 1969; Hebrank and Wright, 1974; Asano et al., 1990; Langendijk and Bronkhorst, 2002), without a method to effectively isolate the influence of the two cues, it remains unclear what spectral features require individualization.

The current work presents a method for decomposing an HRTF into a series of components that are believed to be perceptually separable. With this decomposition, it is believed that the physical features governing localization in the vertical and front-back dimensions reside only in a subset of the resulting components. If such a subset exists, utilizing this decomposition technique should allow more focused efforts in future works designed to identify relevant spectral cues and model localization in these dimensions.

## 2. Materials and methods

### 2.1. Spectral decomposition

**Figure 1:**
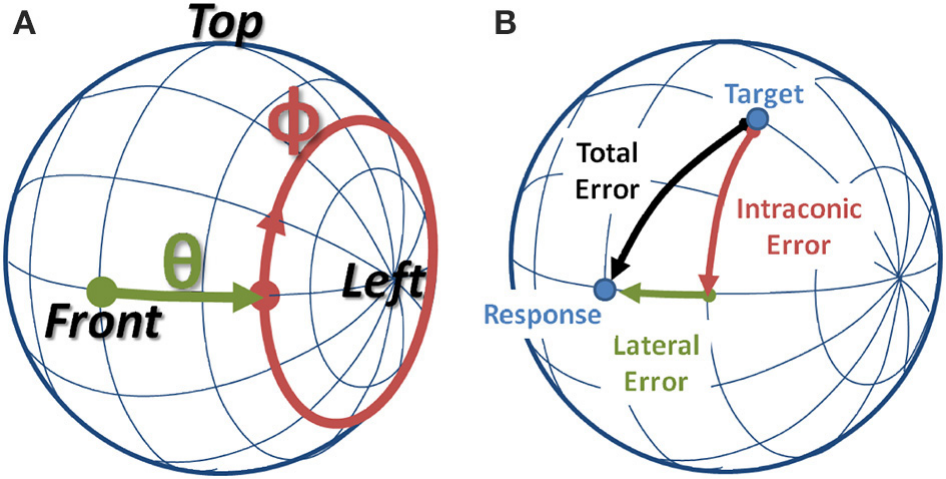
**(A)** Interaural-Polar coordinate system. **(B)** Example of a localization error broken down into lateral and intraconic components.

In order to separately address the cues believed to mediate sound localization, the interaural-polar coordinate system was adopted and employed for both the HRTF decomposition and for depicting the behavioral data. In the interaural polar coordinate system, depicted in Figure 1A, one can define a lateral angle (−90^*o*^ ≤ θ ≤90^*o*^) along the interaural axis, and the intraconic angle (−180^*o*^ < ϕ ≤180^*o*^), where intraconic was chosen to highlight the fact that the parameter approximately describes the angular path along the cone-of-confusion for a given lateral angle. In addition, henceforth, the term “HRTF” will be used to refer to the entire set of spatial filters, while “sample HRTF” will be used to indicate a spatial filter corresponding to a single location.

The spectral decomposition technique requires that sample HRTFs be measured at (or interpolated to) a semi-regular spacing in the interaural-polar coordinate system. For simplicity, it will be assumed that the baseline HRTF was sampled every five degrees in both the lateral dimension, θ_*s*_ = {−90, −85, …, 0, …, 85, 90}, and intraconic dimension, ϕ_*s*_ = {−175, −170, …, 0, …, 175, 180}. First, the average HRTF spectrum across all locations is subtracted from each sample HRTF to create directional spectra. Then, for each lateral angle measured, a lateral spectrum is computed by finding the median spectrum of all the directional spectra measured at that lateral angle. Finally, intraconic spectra are computed by taking the difference between the directional spectrum at each location and the corresponding lateral spectrum.

**Figure 2:**
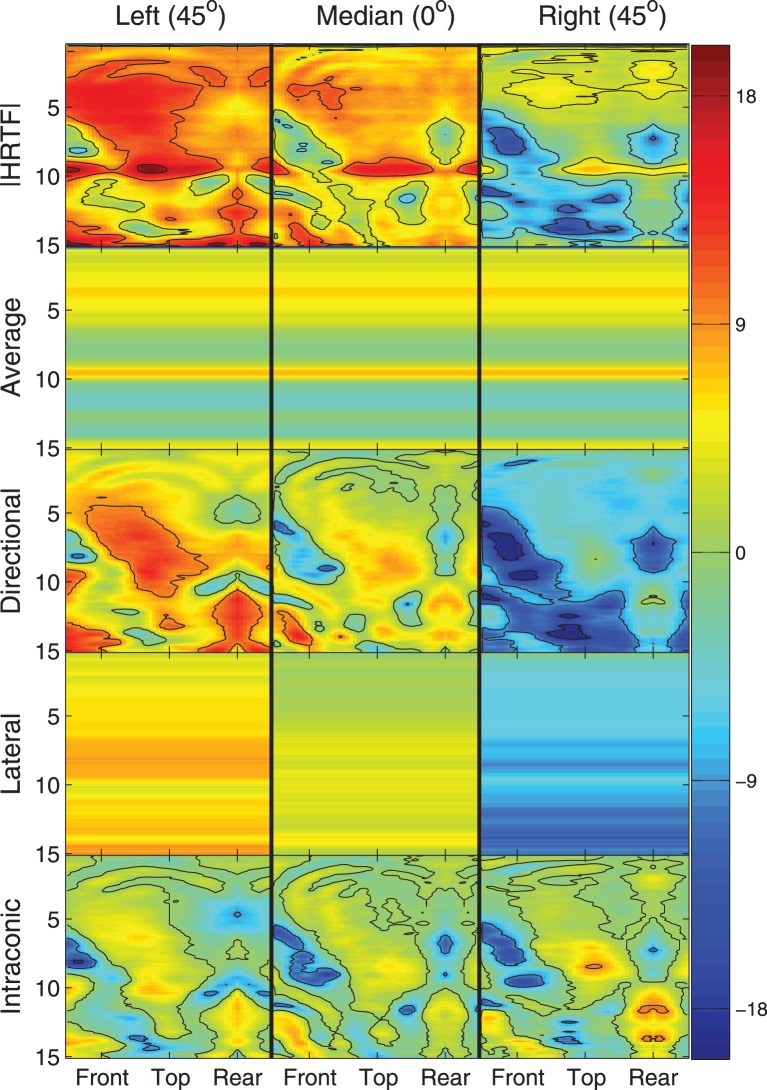
**Illustration of HRTF decomposition into individual components (rows) and three different lateral angles (columns)**. Each panel represents a spectral component around the correspondin cone of confusion as a function of frequency in kHz (ordinate) and intraconic angle (abscissa). Color represents absolute level in decibels.

Figure 2 provides a graphical example of the decomposition stages (rows) for locations along the intraconic dimension at three different lateral angles (columns). In each panel, heat maps are plotted that show the left-ear spectra for a single listener as a function of frequency (ordinate, kHz) and intraconic angle (abscissa, indicated positions are relative to listener). Color indicates the decibel level of each frequency-space bin and contour lines are drawn every 9 dB. This figure illustrates the fact that while the full spectra, the directional spectra, and the intraconic spectra are different for each location, the average spectra and the lateral spectra are constant across all locations and across all intraconic angles of a specific lateral angle, respectively.

The original spectrum at any sampled location can be reconstructed by adding together the average spectrum, the lateral spectrum corresponding to the lateral angle, and the intraconic spectrum from the sampled location. A spatial filter can then be reconstructed by converting the full spectra into the time domain using minimum phase assumptions, and delaying the resulting contralateral impulse response by the interaural time-difference (ITD) value. Alternatively, individual components from one HRTF can be swapped out for the components from a different HRTF measurement before reconstruction to create novel HRTFs constructed with components from two different measurements. In the current study, we examine the importance of having individualized HRTF measurements on a component-by-component basis by constructing HRTFs that have some individualized components and some components from an HRTF measured on a KEMAR acoustic mannequin.

### 2.2. Experimental methods

#### 2.2.1. Subjects

Nine paid listeners (4 males, 5 females) with audiometric thresholds in the normal range (less than 20 dB HL from 150 to 8 kHz) participated in the study over the course of several weeks. All subjects had completed both free-field and virtual localization studies prior to the start of the experiment.

#### 2.2.2. Facility

**Figure 3:**
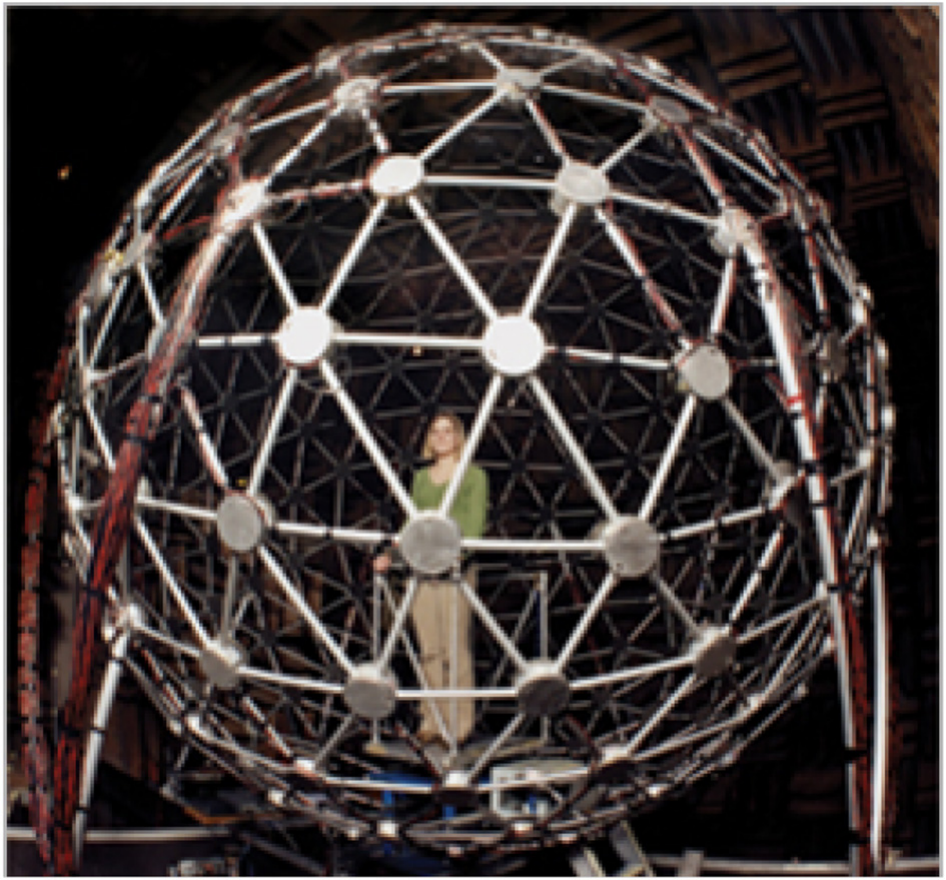
**The auditory localization facility at Wright-Patterson AFB, OH**.

All of the behavioral research was conducted in the Auditory Localization Facility (ALF), located at the Air Force Research Laboratory, Wright Patterson AFB, OH (Figure 3). The ALF consists of a large anechoic chamber with 4-foot fiberglass wedges on all six surfaces and a suspended floor. Inside the chamber is a 7-foot-radius geodesic sphere with Bose loudspeakers positioned at each one of its 277 vertices. The sphere is also outfitted with a 6-DOF ultrasonic tracker (Intersense IS 900) and a cluster of 4 LEDs at the face of each loudspeaker. During measurement and testing, listeners stand on a small platform inside the sphere with their interaural axis aligned vertically with the center of the sphere.

#### 2.2.3. HRTF Collection

For each individual listener and a KEMAR acoustic mannequin, an HRTF was measured at the beginning of the study according to the methods described in Brungart et al. (2009). In short, subjects were outfitted with binaural microphones that blocked off, and sat flush with, the entrance of the ear canal while broadband signals (periodic chirps) were presented from each ALF loudspeaker location and recorded binaurally. A similar process was used for the KEMAR mannequin, but utilized the built-in ear-canal microphones (GRAS 46AO). The resulting recordings were subsequently used to calculate a sample HRTF for each location in the form of 256 Discrete Fourier Transform magnitude coefficients for each ear and a corresponding ITD. ITDs were found by taking the difference in slope of the best-fit lines to the unwrapped low-frequency (300–1500 Hz) phase response of each ear. Magnitude responses were then converted to the decibel scale and decomposed into average, lateral, and intraconic components using the method described in Section 2.1. Headphone (Beyerdynamic DT990) correction filters were also collected for each subject (and KEMAR) using a similar measurement technique (described in Brungart et al., 2009).

#### 2.2.4. Stimuli

During the study, each experimental block consisted of 205 trials. All stimuli within a block were rendered using the same HRTF, which was reconstructed from the listener's individualized HRTF with up to a single component swapped for the corresponding component measured on KEMAR. For example, stimuli in the “Lat” condition were filtered with an HRTF that had been reconstructed with the ITD, average spectrum, intraconic spectrum, and headphone correction filter measured on the current listener, but with the corresponding lateral spectrum taken from a KEMAR HRTF. In each HRTF condition a different component of the listener's individualized HRTF was swapped out for the corresponding KEMAR component; none, the ITD, the average spectrum (Ave), the headphone correction filter (HpTF), the lateral spectrum (Lat), or the intraconic spectrum (IC). Each subject completed two blocks of each HRTF condition, and the presentation order was randomized across listeners. On 90% of the trials, the raw stimulus (i.e., before being filtered with an HRTF) consisted of a 250-ms noise burst, bandpass filtered between 200 and 15 kHz. On the remaining 10% of the trials the same stimulus was extended out to 10 s in duration to allow for exploratory head movements. The presentation order for the stimulus duration was randomized across trials. In a follow-up experiment listeners completed similar blocks with HRTFs constructed from a complete KEMAR HRTF, and a KEMAR HRTF where the IC spectrum was swapped to match the listener's measured IC spectrum.

For all conditions, the virtual stimuli were rendered in real-time using SLAB, a software based virtual acoustic environment rendering system (Miller and Wenzel, 2002). The current implementation of the software allows for real-time head movements of the listener to be incorporated into the virtual rendering, and has been shown in previous studies to support accurate localization when a subject's individualized HRTFs are employed (Brungart et al., 2009).

#### 2.2.5. Procedure

Listeners began the task by donning headphones, a head-tracker and a hand-held tracked wand then pressing a trigger button on the wand. A virtual stimulus was then presented to the listener and they were asked to indicate the perceived location of the stimulus by pointing the wand at the perceived source location, and then pressing a response button on the wand. As the subject pointed the wand, the LEDs on the speaker closest to the direction indicated by the wand were illuminated, creating a dynamic wand-slaved cursor. After the listener responded with a localization judgment, a feedback LED cluster was illuminated at the target location, and the subjects had to acknowledge receipt of the feedback by pressing a wand button that corresponded to the number of LEDs (1–4) used in the feedback presentation. Subsequent trials progressed without a fixed inter-stimulus interval, and started automatically when the subject's head-tracked orientation came within 5° of the horizontal plane and became stationary. Here, stationary implies the head's orientation did not change more than 3° in total angular distance between successive pollings of the headtracker, 1 s apart.

**Figure 4:**
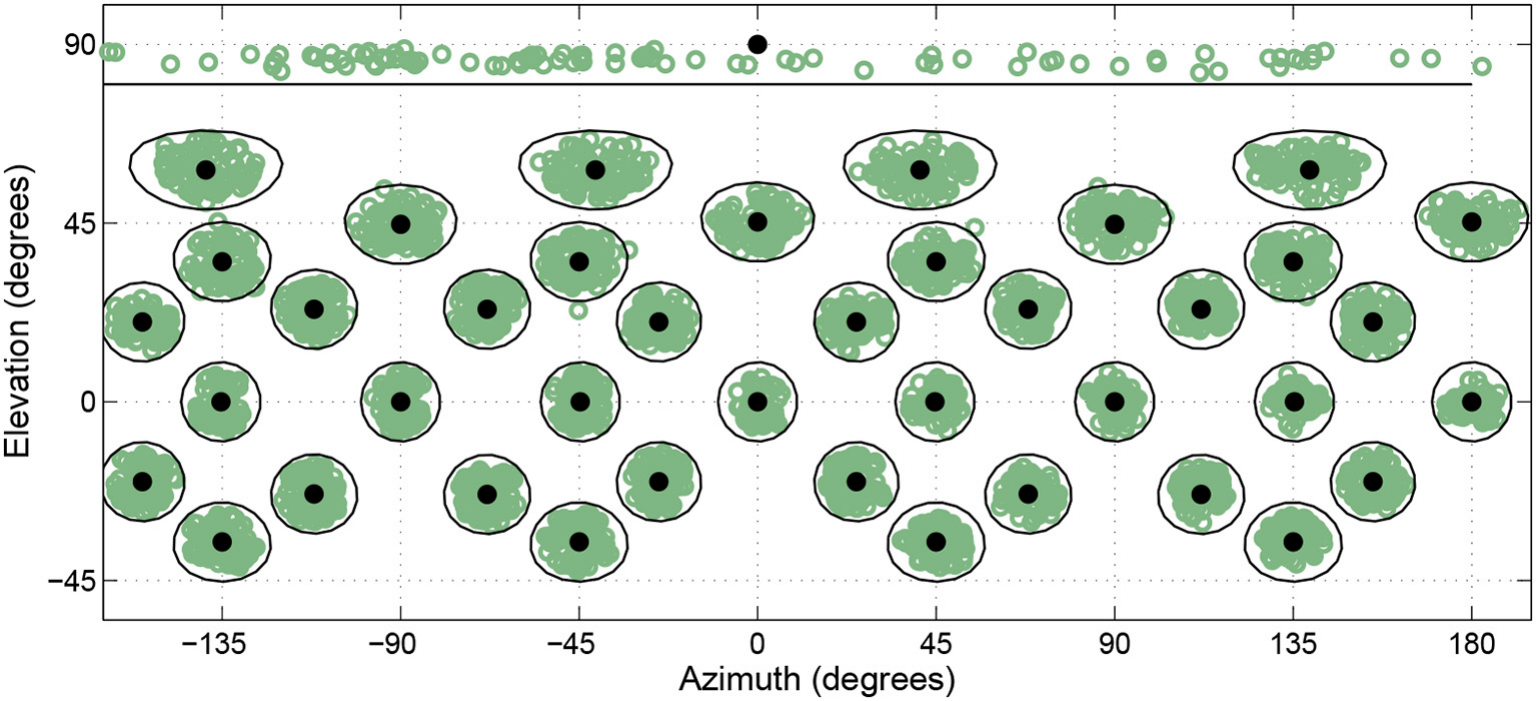
**Actual head-relative target directions (green circles) relative to desired target directions (black circles) for a single subject over the course of the entire study**. Black lines enclose regions within 10° of desired target directions.

On any given trial the desired target direction was 1 of 41 possible head-relative directions distributed throughout 360° in azimuth and from −45° to +90° in elevation. Low elevations were removed due to potential interference with the subject platform. At the time of presentation, the HRTF associated with the actual ALF loudspeaker location closest to the desired target direction was selected and used for rendering the virtual stimuli. By allowing the listeners freedom about what azimuthal direction they were oriented toward at the start of a trial, rather then having them reorient to the same location at the start of every trial, 245 actual loudspeaker locations were used as targets across the course of the experiment even though only 41 different head-relative directions were used as desired target locations. This helped ensure listeners did not learn a specific subset of loudspeaker locations, while allowing for repeated testing of the same small subset of head-relative directions. Figure 4 shows the actual target directions presented over the course of the whole study for a single subject. The black filled circles represent the 41 desired target locations, while the green open circles represent tested target locations. Black rings show a 10° angular distance around each desired location to act as a distance reference under the stretching that occurs toward the poles when the spherical coordinates are plotted on a rectangular grid. As can be seen, the resulting tested target locations end-up tightly clustered and evenly distributed around the desired locations, and almost all tested locations fell within 10° of the desired location.

## 3. Results

**Figure 5:**
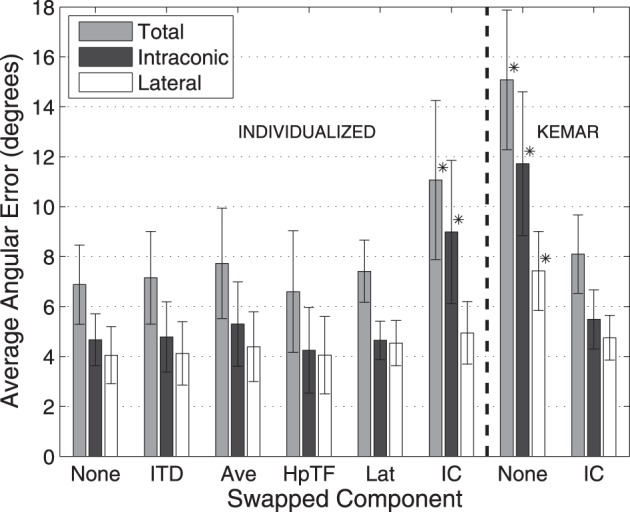
**Average localization errors with 10-s stimuli for each HRTF condition averaged over all subjects**. Errors reported in terms of average total, lateral, and intraconic localization errors. Error bars represent 95% confidence intervals. ^*^Result is statistically different from baseline (indicated in text) (p < 0.005, paired *t*-test).

**Figure 6:**
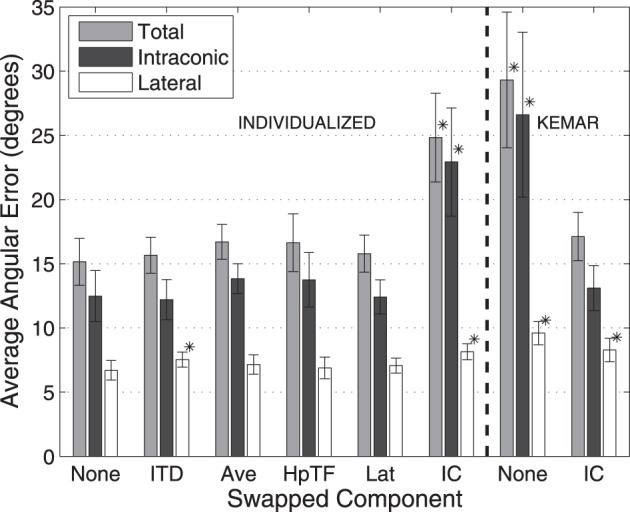
**Average localization errors with 250-ms stimuli for each HRTF condition averaged over all subjects**. Errors reported in terms of average total, lateral, and intraconic localization errors. Error bars represent 95% confidence intervals. ^*^Result is statistically different from baseline (indicated in text) (*p* < 0.005, paired *t*-test).

Figures 5, 6 show average angular errors computed over subject and target location. Localization errors are broken down in terms of the total angular, intraconic, and lateral components (depicted in Figure 1B), and plotted as separate color-coded bars. Each group of bars to the left of the line, labeled “Individualized” represent the first set of conditions in which isolated components (indicated on the abscissa) of the listener's individualized HRTF were swapped out for the corresponding KEMAR component. The two groups of bars to the right of the line, labeled “KEMAR,” represent the two additional conditions in which a full KEMAR HRTF (None), or a KEMAR HRTF with the IC component for the listener's individualized IC component, were used. For example, INDIVIDUALIZED-None is a fully individualized HRTF and KEMAR-None is a full KEMAR HRTF. In all conditions, error bars represent 95% confidence intervals for the means, and asterisks indicate a statistically significant difference (*p* < 0.05) from the baseline condition (Individualized-None) in a paired *t*-test.

The left side of Figure 5 shows the average localization results for the 10-s stimuli for the first experiment. In this condition the stimuli were long enough in duration to allow for exploratory head-motion which likely accounts for the fact that no difference in average angular error was seen when any of components of the HRTF, except the IC component, were swapped. The largest total angular error for any of the individualized HRTF conditions occurred when the IC component was swapped with KEMAR, and resulted in a significant difference in terms of total angular error. As expected most of this error was an increase in intraconic error relative to the none condition (black bars). In contrast, switching out other individualized components resulted in only negligible changes (within 1°) in lateral error.

The left side of Figure 6 utilizes the same format for representing the average results for the 250 ms stimuli. Here, localization errors across all conditions are generally about twice as large as the corresponding conditions with 10-s stimuli (note the change in scale of the vertical axis). This is likely due to the fact that the 250-ms stimuli are too brief to allow listeners to utilize exploratory head movements. As seen in all HRTF conditions this also leads to a larger amount of the total error occurring in the intraconic dimension. Again, there was significant increase in the amount of total angular error when the IC component of the individualized HRTF was swapped out for the KEMAR IC component (25°) compared to the Individualized-None condition (15°), similar to the results with longer stimuli. The results also indicate a significant difference in the lateral error between the None and ITD conditions, as well as between the None and the IC condition, though the overall magnitude of the difference remains quite small (1°–2°).

Based on the results of the initial experimental conditions, two additional conditions were run to investigate how the earlier results compared to performance with a full KEMAR HRTF, and whether performance with a KEMAR HRTF could be improved significantly by swapping out only the IC component for the subject's own. Results from those two conditions are represented to the right of the dashed line in Figures 5, 6. Not surprisingly, the full KEMAR HRTF condition led to the worst performance for all three types of error with an average of about 15° total angular error with the 10-s stimuli, and approximately 28° for the 250-ms stimuli. While significantly worse than the Individualized-None condition, this condition does not appear to be significantly different from the Individualized-IC condition. In contrast, when the IC component of the KEMAR HRTF was replaced with the listener's own IC component, performance improved to the level seen with a fully-individualized HRTF (i.e., the individualized-none condition) for both stimulus durations and error types, with the exception of the lateral error with the 250-ms stimulus.

**Figure 7:**
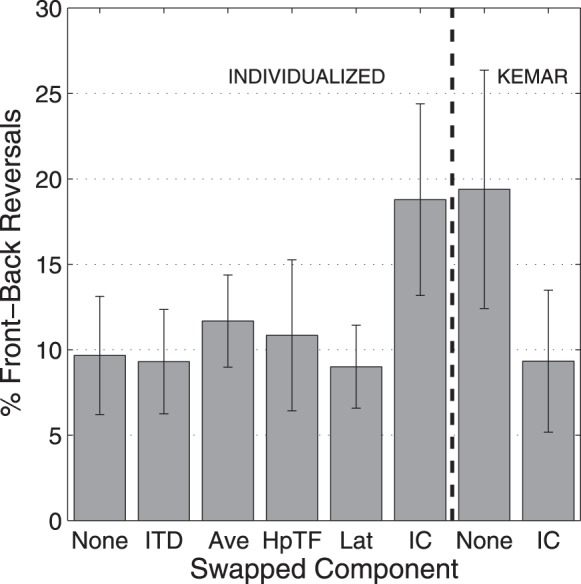
**Percentage of front-back reversals with 250-ms stimuli for each HRTF condition averaged over all subjects**. Error bars represent 95% confidence intervals.

A common occurrence when using virtual audio with non-individualized HRTFs is a large increase in the rate of front-back reversals, trials in which virtual sound sources are perceived to be in the opposite front-back hemisphere to the target location. Figure 7 shows the percentage of trials in which a front-back reversal occurred for the 250-ms stimuli, averaged over subjects. Here, all of the conditions in which there was an individualized IC component resulted in front-back reversals on about 10% of the trials, while the two conditions with a KEMAR IC spectral component resulted in front-back reversals on 20% of the trials.

**Figure 8:**
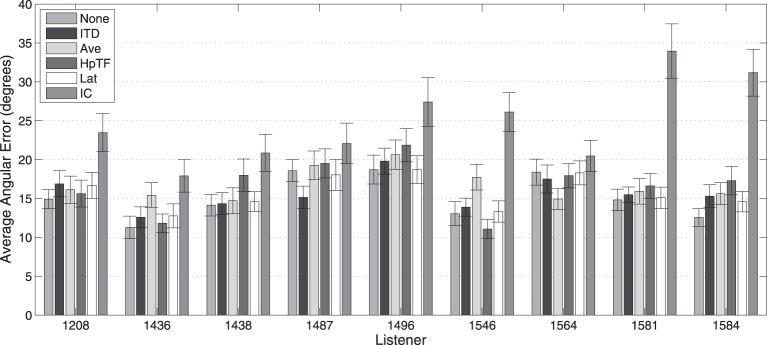
**Average total absolute localization error with 250-ms stimuli for each HRTF condition by subjects**. Error bars represent 95% confidence intervals.

Average localization results for the 250-ms stimuli for each subject from the first experiment are shown in Figure 8. Performance is seen to vary considerably between listeners and across the different HRTF conditions. In the baseline condition, in which no individualized components were swapped for KEMAR components (None), the best total angular error (11°) was achieved by listener 1436, while the worst performer (19°) was listener 1496. Consistent with the average results, all listeners had the worst performance in the IC condition where the listener's own intraconic spectra were replaced with those of KEMAR; however, this modification seemed to hinder some listeners (e.g., 1581) more than others (e.g., 1564).

It is important to note, that although feedback about the target's location was provided on every trial, no significant learning effects were observed. When analyzed separately the first and fifth quintile in each block showed at most 4° of improvement in average angular error, and all of the HRTF conditions exhibited a similar trend across quintiles. In other words, the differences between HRTF conditions in the average results presented above were consistent with the differences observed in quintile averages.

## 4. Discussion

Overall, the localization results agree well with published results for similar experiments using virtual stimuli both from our lab (Brungart and Romigh, 2009; Brungart et al., 2009; Romigh, 2012), and other laboratories (Wenzel et al., 1993; Bronkhorst, 1995; Middlebrooks, 1999b). In fact, in a recent meta-analysis of combined data from more than 82,000 trials collected across 161 listeners in five different laboratories, Best et al. (2011) showed a free-field localization performance of 15.6° total angular error for brief sounds, which corresponds well with the virtual performance seen in the current study with the fully individualized HRTFs. These results suggest that the baseline virtual representation was adequate to preserve all of the relevant localization cues.

Most interesting, the results indicate that the IC spectral component is the component of the HRTF that is most important to maintain virtual localization accuracy comparable to performance with fully individualized HRTFs (and potentially free-field sources). This conclusion comes from the results of both experiments which, taken together, showed that differences between performance with a fully individualized HRTF and a full KEMAR HRTF could be diminished by swapping only the IC component. What this means for future work is that studies focusing on the differences between the HRTFs of individual subjects can be focused on a single component of the HRTF. Moreover, in combination with the previous discussion point, studies geared toward modeling localization in the intraconic dimension can focus their analysis toward only the physical cues contained in the IC component.

The negligible difference seen in localization performance when the other individualized components were replaced with KEMAR equivalents suggests that, for most subjects, generalized values for these components are sufficient for maintaining localization accuracy. Relating the behavioral results back to the anthropometric cause of these cues may suggest that, in terms of acoustical influence, anthropometric properties like head-size, which directly affects the ITD and lateral spectral component (Algazi et al., 2002), may be more consistent across subjects than the pinna shapes that are responsible for the contours of the IC spectral component (Algazi et al., 2001). Conversely, the differences may result from the non-linear nature of the mapping between spectral cues and intraconic location. In other words, a small change to the ITD or lateral spectrum will likely result in a perceptual image near the original, while it is much less predictable where a stimulus with a small spectral modification might be perceived spatially.

The lack of effect seen when swapping out the headphone correction (HpTF component) or the spectral average component suggests that these effects, which in some cases caused severe changes to the resulting HRTF spectrum, are ignored or compensated for when making a localization judgment. Since both of these components would have been consistent for every trial within each block, it is likely that their effects were incorporated into the listener's internal representation of the source spectrum, and therefore treated as directionally uninformative. It is important to note that despite their lack of effect on localization, initial testing by the authors confirmed that very noticeable timbrel differences were apparent when these components were exchanged, which may be of consequence for some types of auditory displays.

### Conflict of interest statement

The authors declare that the research was conducted in the absence of any commercial or financial relationships that could be construed as a potential conflict of interest.
